# Crossing the threshold: can outcome data from food challenges be used to predict risk of anaphylaxis in the community?

**DOI:** 10.1111/all.12997

**Published:** 2016-12-13

**Authors:** P. J. Turner, B. K. Wainstein

**Affiliations:** ^1^ Section of Paediatrics (Allergy and Infectious Diseases) & MRC and Asthma UK Centre in Allergic Mechanisms of AsthmaImperial College LondonLondonUK; ^2^ Discipline of Child and Adolescent HealthSydney Medical SchoolUniversity of SydneyAustralia; ^3^ Department of Immunology and Infectious DiseasesSydney Children's HospitalSydneyNSWAustralia; ^4^ School of Women's and Children's HealthUniversity of New South WalesSydneyNSWAustralia

There is increasing interest in using data from oral food challenges (OFC) performed under medical supervision to assist in allergy risk management, both in industry (allergen risk management) 1 and in the clinical management of the allergic individual 2. Data relating to the minimum eliciting dose (MED) needed to trigger symptoms can inform the need for precautionary allergen labelling (PAL) on food 1. However, given that ‘zero risk’ for food‐allergic individuals is not considered to be a realistic proposition 3, it is also important to consider the severity of symptoms which might be experienced: for example, a pragmatic approach, using a level of exposure that causes only minimal, transient oral symptoms in under 1% of the food‐allergic population, may be acceptable to guide the use of PAL 4. However, there is often an assumption that individuals with a lower MED (i.e. who react to a lower doses of allergen) are at a greater risk of anaphylaxis 5.

Much of this seems logical, although there are clearly limitations: we do not yet understand the inherent variability and reproducibility of an individual's allergen threshold, although useful data will be generated by the TRACE Peanut Study (NCT01429896). The OFC scenario is very different from ‘real‐life’ exposure: consumption of incremental doses of allergen at regular intervals (typically every 15–30 min) during OFC may induce a transient desensitizing effect leading to an overestimation of the MED 6. Moreover, OFC are usually terminated at the onset of objective and often mild symptoms for safety reasons. The literature indicates a median time for onset of severe symptoms following exposure to food allergen in the community of 10–30 min 7, 8; in contrast, using an extended dosing interval of 2 h, Blumchen et al. reported a median time to objective symptoms of 55 min (range 5–210 min) 9. Whether this disparity might also be due to a transient desensitizing effect with longer dosing intervals is unclear 10. For these reasons, a typical OFC regimen may overestimate MEDs, with many patients reacting to *not* the immediate preceding dose but a dose given 1–2 h beforehand.

## What is the relationship between dose of exposure and reaction severity?

In much the same way that previous reactions do not predict the severity of future reactions 2, symptoms at in‐hospital OFC do not reflect reactions occurring in the same individuals in the community 11, and vice versa 12. Presumably, this is due, at least in part, to the incremental dosing regimen used for OFC, allowing (at least in theory) for the challenge to be halted prior to onset of more severe symptoms. In contrast, allergic individuals consume (relatively) larger doses of allergen more rapidly in the community, before becoming aware of any symptoms. Therefore, the relationship between dose of exposure and resulting symptoms is unclear. One might expect a dose–response to exist (as is typical for most agonist/receptor interactions), with more severe symptoms being associated with higher levels of exposure. However, the data are seemingly contradictory: severe reactions at OFC have been reported at all levels of allergen exposure, down to milligram quantities 9, 13. Furthermore, data from OFC studies (which have not excluded individuals with prior anaphylaxis) suggest that individuals with prior anaphylaxis do not react to lower doses of allergen than those without 9, 12, 14, 15. The implication is that individuals who have previously reacted to very small levels of exposure are *not* more at risk of anaphylaxis, something which seems counter‐intuitive to clinical experience 16 and practice 5.

We (BKW) have previously reported a cohort of children who underwent OFC to peanut 17. In contrast to other studies, OFC were not terminated at onset of mild symptoms but allowed to progress. Twenty‐seven children experienced positive reactions. Anaphylaxis was provoked in 21 (78%); in 13 cases, this was due to further allergen ingestion following initial mild symptoms. Of note, 6/27 children (22%) did not develop anaphylaxis despite completing the challenge (cumulative dose of 2.9 g peanut protein, equivalent to approximately 14 peanuts).

We have conducted a further analysis of this cohort, with reference to both the dose causing any symptoms versus that triggering anaphylaxis. We have also enriched the cohort with peanut‐allergic children undergoing double‐blind, placebo‐controlled food challenges according to international consensus 10 as part of a desensitization study (BOPI study, NCT02149719), who also ingested further peanut following initial mild objective symptoms (which did not meet the stopping criteria for OFC). Children who did not experience objective symptoms were excluded from analysis. Both studies received local ethical approval, and written informed consent was obtained prior to OFC. The demographics of these cohorts are described in Table 1.

**Table 1:** Patient characteristics

Characteristic	Sydney Cohort	BOPI Cohort
No. of patients included	21	16
Age at challenge (mean ± SD)	5.5 ± 3.7 years	12.7 ± 2.5 years
SPT wheal size to commercial peanut extract (median, interquartile range)	11 mm (6.5–15.5 mm)	9 mm (6–23 mm)
Peanut‐specific IgE (kUA/l)	20.5 (0.86–>100)	33.9 (0.62–>100)
IgE to rAra h 2 (kUA/l)	Not done	13.2 (0.21–>100)
Gender, no. (%)		
Male	17 (81)	10 (62)
Female	4 (19)	6 (38)
Other atopy, no. (%)		
Asthma	7 (33)	13 (82)
Allergic rhinitis	7 (33)	14 (88)
Eczema	13 (62)	7 (44)
Other food allergy	6 (29)	8 (50)
Outcome of OFC no. (%)		
Anaphylaxis	21 (100)	16 (100)
As initial symptom	3 (14)	2 (13)

SPT, skin prick test; OFC, oral food challenge.

## We have identified 3 patterns of reactivity

1. Those who experience nonanaphylactic symptoms despite consuming the top dose (Fig. 1). Whether these subjects would go on to experience anaphylaxis with further ‘supra‐threshold’ allergen exposure beyond the 4–5 g protein dose recommended by international consensus 10 is unclear; at least one case report indicates this is a possibility 18.

**Figure 1:**
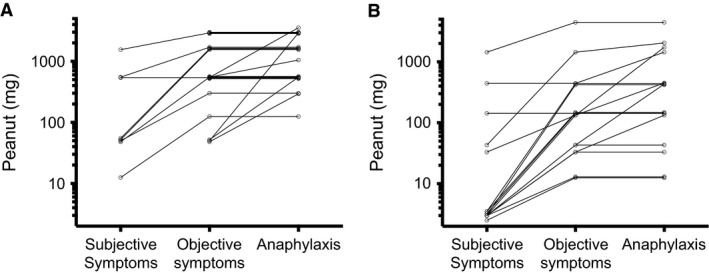
Patterns of reactivity seen during OFC to peanut in allergic children. (A) Open OFC (*n* = 21) resulting in anaphylaxis, as previously reported 17. (B) Double‐blind placebo‐controlled food challenges (*n* = 16) resulting in anaphylaxis as part of the BOPI study. Each line represents a single individual undergoing OFC.
2. Individuals who experience initially mild symptoms, but develop anaphylaxis with further exposure.
3. Allergic individuals who develop anaphylaxis as their initial symptom (often without preceding subjective symptoms), a phenomenon which can occur at all dosing levels.

**Figure 2:**
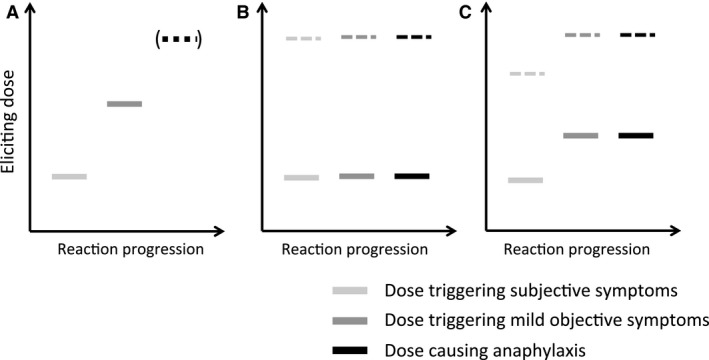
Different patterns of clinical reactivity seen in OFC. (A) Many individuals will experience initially subjective symptoms, with objective symptoms appearing with further doses. Typically, the OFC is halted at this stage; however, if the OFC is continued, anaphylaxis will develop in most individuals 17. (B and C) Others will experience anaphylaxis as their first objective symptom: either at a dose of allergen exposure with no preceding subjective symptoms (B), or with prior subjective symptoms (C). Note that anaphylaxis can occur at all levels of exposure (both at low levels of allergen exposure, represented by the solid bars, and higher doses indicated by dotted lines).

We interpret these data as suggesting that individuals may have a threshold of reactivity for both any symptoms and a further threshold for symptoms of anaphylaxis (Fig. 2). In some, these two are very similar: such individuals will experience anaphylaxis as their initial symptom at OFC, with no apparent relationship between dose and severity. In others, there may be a significant difference between the two: in these subjects, a dose–severity relationship will be seen. However, at a group or population level, this relationship is diluted by individuals in the former category, and those who do not experience anaphylaxis due to the dose‐limiting regimen used for OFC. Thus, while for most individuals it is likely a dose–severity relationship exists, this may not be seen at a population level.

## What conclusions can be drawn from this model?

A major premise of OFC is the assumption that the procedure effectively dose‐limits the risk of anaphylaxis. Our analysis implies that this probably holds true at an individual level, for most allergic individuals. It is important to consider how cofactors or augmenting factors 2, 19 may affect both the threshold/likelihood of any reaction, as well as the threshold triggering anaphylaxis. This clearly is difficult – to intentionally induce anaphylaxis presents both ethical and safety issues. Where the primary purpose of OFC is to determine the MED (rather than confirm clinical reactivity or assess response to immunomodulation), consideration must be given to increasing the time interval in between doses. Fortunately, current attempts to use MEDs in improving allergen risk management mostly relate to ED01/ED05 levels (eliciting dose to provoke a reaction in 1% or 5% of the allergic population, respectively). This dosing level is generally equivalent to the first or second dose given during OFC, so overestimation of eliciting doses in the remaining 99%/95% of the population is unlikely to be a significant concern, something supported by the literature 20. Furthermore, low‐dose, ‘single‐dose’ challenge (at the ED05) levels are being used to further verify existing data 21.

The current PRACTALL recommendation for a seven‐ to eight‐dose OFC protocol can be time‐consuming in clinical practice. Clinicians may be tempted to use a lower number of doses in a lower risk, routine clinical setting where OFC are used to rule out clinical allergy (see example in Table 2). Such a protocol can result in some patients being given a higher dose than they might receive under a PRACTALL regimen, thus increasing the risk of more a severe reaction (a risk which might be greater if existing population dose distributions are overestimates).

**Table 2:** Typical OFC protocol for peanut according to PRACTALL Consensus 10, and how this compares to an abbreviated protocol derived from the available dose‐distribution data 1

PRACTALL dosing (mg peanut protein)	Proportion of peanut‐allergic population reacting to this level of exposure, %	Abbreviated protocol (mg peanut protein)
3	~8	
10	~20	10
30	~30	
‐	~40	60
100	~50	
300	~65	300
1000	~80	1000
3000	~95	3000

Finally, the applicability of OFC data to inform the relationship between dose and severity in real‐life community exposures will depend on the quality of the OFC protocol used, how representative the individuals challenged are to the wider allergic population (many historical studies excluded individuals with a history of anaphylaxis) and the completeness of the data set analysed. Despite a wealth of OFC data being reported in the literature, the quality of these data is not always apparent. In many cases, individual threshold data are not in the public domain; as a result, attempts to analyse the dose–severity relationship can be misleading. Zhu et al. analysed MEDs in the literature relating to individuals undergoing OFC and compared these to the severity of symptoms experienced. They reported that peanut‐allergic individuals experiencing severe reactions had significantly higher MEDs than those with more mild reactions 22. However, many of the series analysed only reported MEDs for those with anaphylaxis or systemic reactions, and not for more mild reactions. As a result, over 40% of the MEDs reported in the source studies were not available for analysis. The resulting skewing of data, together with the likelihood that MEDs derived from OFC data for anaphylactic reactions may overestimate the true eliciting dose (for the reasons highlight above), almost certainly resulted in an unintended distortion of the available data.

Given the inherent risks of OFC, it is time that data relating to MEDs (particularly that arising from publicly‐funded research) are made freely available through a data‐sharing scheme, a scenario being proposed by leading medical journals 23. This would allow the community to better determine the relationship between dose and severity at OFC, and judge the applicability of this data to improve the management of allergic patients in the community.

## Funding

PJT is in receipt of a Clinician Scientist award funded by the UK Medical Research Council (reference MR/K010468/1) and is supported by the National Institute for Health Research (NIHR) Biomedical Research Centre based at Imperial College Healthcare NHS Trust and Imperial College London. The views expressed are those of the author(s) and not necessarily those of the NHS, NIHR or the Department of Health. PJT is a member of an industry‐sponsored ILSI expert group on predicting reaction severity.

## Conflicts of interest

All authors declare no conflict of interest.

## Author contributions

This article was co‐written by PJT and BKW, who both approved the final version.
